# Acquired Factor X Deficiency in Patients With Primary Light Chain Amyloidosis

**DOI:** 10.1177/2324709619832332

**Published:** 2019-04-04

**Authors:** Siroj Dejhansathit, Attaya Suvannasankha

**Affiliations:** 1 Indiana University School of Medicine, Indianapolis, IN, USA

**Keywords:** factor X deficiency, blood coagulation disorders, AL amyloidosis

## Abstract

Acquired factor X (FX) deficiency is a rare but serious complication of primary amyloidosis, presumably caused by the binding of amyloid proteins to the clotting factors. The prolonged prothrombin time, partial thromboplastin time, and low FX level, which are correctable by mixing study, are the disease hallmarks. An immediate goal of care is to stop bleeding. Clotting factor replacement requires close monitoring of coagulogram and FX levels due to varying FX clearance among patients. High-purity FX is currently approved for hereditary FX deficiency and has been successfully used in some acquired FX deficiency cases. Ongoing bleeding risk complicates the treatment decision. Novel therapies yielding rapid and deep response reduce amyloid protein production and improve long-term outcome.

## Case Series

### Case 1

A 42-year-old woman presented to the emergency department with acute abdominal pain and was diagnosed with a hemoperitoneum from a ruptured ovarian cyst. The laparoscopic evaluation was complicated by continuous bleeding. Additional workup confirmed a partial thromboplastin time (PTT) of 42 seconds (normal 20-36 seconds), which was corrected by mixing study, suggesting factor deficiency. Factor X (FX) activity was 11%, while others were normal. She required multiple units of fresh frozen plasma (FFP) and aminocaproic acid for bleeding control. FX activity did not significantly improve after FFP infusion but improved transiently with prothrombin complex concentrates (PCC). However, to maintain a FX activity over 20%, a higher dose and more frequent infusions were required. Serum protein electrophoresis showed IgG kappa monoclonal protein of 0.4 g/dL, and serum-free kappa and lambda light chain levels were 14.5 and 72.2 g/dL, respectively. Bone marrow aspiration and biopsy showed 3% kappa-restricted plasma cells. Congo red staining of bone marrow was negative, but rectal biopsy tissue was positive. Immunofluorescent staining confirmed kappa-restricted amyloid protein. Amyloidosis of the kidney was also suspected based on 3 g/24 h proteinuria, though kidney biopsy was not performed. She had no hepatosplenomegaly. Troponin, pro-BNP levels, and echocardiogram were normal. The patient had ongoing bleeding complications including heavy periods requiring uterine ablation and intermittent lower gastrointestinal bleeding. She started induction therapy using bortezomib, cyclophosphamide, and dexamethasone regimen (CyborD). She developed grade 2 painful sensory neuropathy and only achieved marginal response after 3 cycles of therapy. Her FX activity remained between 10% and 15%. She proceeded to stem cell collection, followed by high-dose melphalan and autologous stem cell transplantation. During the myelosuppressive period, she received PCC and platelet transfusion to keep the platelet level over 20 × 10^9^/mL. She achieved complete remission as evidenced by the normalized serum lambda light chain and correspondingly had a gradual improvement of FX level.

### Case 2

A 67-year-old man presented with an ankle swelling and cutaneous purpura. Additional workup showed 7 g/dL of albuminuria, and kidney biopsy confirmed lambda-restricted amyloid protein deposition. The patient had a bleeding complication from kidney biopsy, which led to additional investigations confirming a low serum FX activity (20%). Splenomegaly was noted on the computed tomography of the abdomen. He had no bleeding history but reported easy bruising for the past 3 months. Serum protein electrophoresis showed 1.0 g/dL of IgG lambda monoclonal protein. Serum-free kappa and lambda light chain levels were 18 and 34 g/dL, respectively. Bone marrow aspiration and biopsy confirmed 5% of lambda-restricted plasma cells. The patient opted against stem cell transplantation and underwent induction therapy with the bortezomib-based regimen. He achieved complete hematologic remission. Serum FX level improved but remained low at 35%. The patient was in an auto accident resulting in a splenic rupture. He underwent emergent splenectomy and required red blood cell transfusion during the procedure. The serum FX level improved to 52% within 1 month after the surgery.

## Discussion

Bleeding complications are common in patients with primary amyloidosis (AL amyloidosis; AL). Multifactorial causes include vasculopathy due to amyloid protein deposition, production defect of clotting factors due to liver failure, and, less commonly, clotting factor deficiency due to adsorption of clotting factors by amyloid protein.^1^ This case series illustrates an unusual presentation of acquired FX deficiency in systemic amyloidosis. FX deficiency is the most common coagulation factor deficiency in amyloidosis, occurring in 6.3% to 14% of patients.^2^ AL is the only described cause for acquired isolated deficiency of FX.

FX involves both the intrinsic and extrinsic pathways of coagulation cascades^3^; therefore, the prolonged prothrombin time and PTT, and low FX levels, corrected by mixing study, are clues to FX deficiency rather than the presence of clotting inhibitors.^4^ Acquired FX deficiency in patients with primary amyloidosis is secondary to the adsorption of FX to amyloid fibrils in the reticuloendothelial system leading to a shortened half-life.^5,6^ While in congenital FX deficiency bleedings tend to occur only in severe cases with FX level <10% (0.01 IU/mL), patients with AL-related FX deficiency can develop bleeding complications at higher FX levels. In our case series, spontaneous bleeding occurred when FX levels were between 10% and 25%. The above-mentioned evidence highlights the more complicated bleeding diathesis in primary amyloidosis where other variables such as vascular integrity, clot lysis rate, platelet count, and function may also play a role.^7^ In a series of acquired clotting deficiency in AL amyloidosis, serious bleeding can occur in up to 56% of patients.^1^ This includes excessive bleeding after invasive procedures, intracranial, umbilical cord, joint, and muscle bleeding.^3^

For acute bleeding, correction of factor deficiency is the primary goal. FFP, PCCs, activated PCCs, recombinant factor VIIa (rFVIIa), and most recently available high-purity FX have been successfully used. FFP, PCCs, and activated PCCs have a varying amount of FX concentration (Table 1). Advantages of PCCs versus FFP include more rapid correction of FX deficiency due to higher FX concentration in PCCs; greater increase in other clotting factors, which may benefit patients with acquired deficiency of other clotting factors in addition to FX case of combined clotting factor deficiency; fewer complications secondary to fluid overload; and shortened preparation time since PCCs do not need to be thawed and do not require blood typing. While recommended dosing of these products are based on factor IX concentration in the product, the dose required for acquired FX deficiency is not known, and therefore, the measurement of FX activity post infusion is needed. High-purity FX concentrations (Factor X P Behring; CSL Behring, King of Prussia, PA; and Coagadex; Bio Products Laboratory, Elstree, England) have recently received an Food and Drug Administration approval for the treatment of hereditary FX deficiency, but not yet for acquired FX deficiency, despite its successful application in a small case series. It is not available locally; therefore, its use may be limited to large medical centers.

**Table 1.** Factor Compositions in the Prothrombin Complex Concentrates (PCC), as Ratio to Factor IX.

	FII	FVII	FIX	FX	Dose^a^
*3-Factor PCC*					
Bebulin (USA)	120 units	13 units	100 units	139 units	25-35 units/kg (minor bleed); 40-55 units/kg (moderate bleed); 60-70 units/kg (serious bleed)
Prothrombinex HT (Australia)	100 units	—	100 units	100 units	25-50 units/kg
Cofact (Europe and UK)	~75 units	~25 units	100 units	~75 units	25-50 units/kg
Profilnine SD (USA)	148 units	11 units	100 units	64 units	25-50 units/kg
*4-Factor PCCs*					
Beriplex (UK), KCentra (US)	128 units	68 units	100 units	152 units	25-50 units/kg
Prothromplex T (Austria)	100 units	85 units	100 units	100 units	25-50 units/kg
Octaplex (UK and Europe)	44-152units	36-96 units	100 units	72-120 units	25-50 units/kg

a Dose of PCC product is based on factor IX component.

Due to the variation in the adsorption and clearance of FX by amyloid protein, it is mandatory to monitor and adjust the frequency as well as the concentrations of these products based on the levels of PTT as well as FX activity after each transfusion, as shown in Case 1. At present, the optimal target after replacement for the FX levels in patients with primary amyloidosis is not known; however, it can be derived from that of patients with congenital FX deficiency: 10 to 15 IU/dL for minor bleeding and >50 IU/dL for major bleeding, trauma, or surgery.^8^ Physician needs to exert extra caution for volume overload particularly in AL amyloid patients with cardiac involvement when a large volume of factor replacement is used.

Other therapies with reported use in acquired FX deficiency include activated rFVIIa. rFVIIa binds to tissue factor exposed on injured tissue to activate factor IX, factor X, and platelets.^9^ In addition, high concentrations of factor VIIa can directly activate platelets and local thrombin formation.^10^ rFVIIa may be effective in treating persistent skin and muscle bleeding, and intraoperative bleeding.^11^ However, successful thrombin generation requires activation of FX downstream of FVII, and rFVIIa may be ineffective in cases of severe FX deficiency. Both rFVIIa and PCC increase the risk of thrombosis, particularly in patients with nephrotic proteinuria whose thrombotic risk is already high; therefore, close observation for thrombosis is warranted.

Plasma exchange has been used to remove clotting inhibitors.^6^ Plasma exchange with FFP allows a transient replacement of FX while avoiding volume overload, but the benefit is short-lived due to adsorption of the replaced FX by the amyloid protein. Central venous access needed for plasma exchange increases bleeding risk. Antithrombotic agents including aminocaproic acid or tranexamic acid do not directly correct FX deficiency but provide clot stabilization. They can be used together with clotting factor replacement in serious bleeding and can be adequate to use in mucosal bleeding particularly in an outpatient setting.

A majority of FX adsorption is believed to occur within the reticuloendothelial system. Rapid correction of FX levels has been reported post-splenectomy and extensive amyloid deposition seen in the removed spleen.^5^ However, splenectomy carries a high bleeding risk, which can be attenuated by factor replacement. In Case 2, the patient had a mild FX deficiency and had already responded to induction therapy. Splenectomy yielded a rapidly improved FX activity.

The long-term outcome of amyloidosis-related FX deficiency depends on the response to treatment of primary amyloidosis. High-dose chemotherapy and stem cell transplantation have been a standard of care for young and fit patients with limited organ involvement. Patients with cardiac involvement or extensive organ involvement are typically treated with combination chemotherapy. Newer agents including proteasome inhibitors and immunomodulatory agents yield rapid response, while having low toxicities, and have been incorporated into AL amyloidosis algorithms at diagnosis and relapse. FX activity improvement may lack behind hematologic response, so the risk of bleeding continues long after hematologic remission. Serial FX levels and factor replacement for bleedings and prophylaxis prior to invasive procedures are recommended.^12^ Patients are recommended to wear medical alert bracelets highlighting their underlying bleeding disorder, in case of bleeding emergency.

In summary, we report the case series of acquired FX deficiency and review the current standard of care, where early recognition and factor replacement is lifesaving.
